# Synthesis and crystal structure of tri­carbonyl­chlorido­{1-[(pyridin-2-yl­methyl­idene)amino]­adamantane}rhenium(I)

**DOI:** 10.1107/S205698901601255X

**Published:** 2016-08-09

**Authors:** Jorge Jimenez, Indranil Chakraborty, Pradip Mascharak

**Affiliations:** a Department of Chemistry, University of California Santa Cruz, CA 95064, USA

**Keywords:** crystal structure, rhenium complex, *fac*-Re(CO)_3_, lipophilicity, adamantane motif

## Abstract

The title compound, [ReCl(pyAm)(CO)_3_], where pyAm is 1-[(pyridin-2-yl­methyl­idene)amino]­adamantane (C_16_H_20_N_2_), was synthesized from the reaction of [ReCl(CO)_5_] and pyAm in an equimolar ratio. The Re^I^ atom resides in an octa­hedral C_3_ClN_2_ coordination sphere. The Re—C bond *trans* to the chloride ligand is noticeably longer compared to the other two Re—C distances. Weak C—H⋯Cl hydrogen-bonding inter­actions consoldiate the packing of the mol­ecules. In this design, the pyAm ligand was employed due to its well-known pharmacokinetic properties.

## Abstract 2

The compound [ReCl(pyAm)(CO)_3_], where pyAm is 1-[(pyridin-2-yl­methyl­idene)amino]­adamantane, comprises an Re^I^ atom with an octa­hedral C_3_ClN_2_ coordination set.

## Chemical context   

The diverse photophysical and photochemical properties of tri­carbonyl­rhenium(I) complexes make them invaluable for a range of applications, such as light-emitting devices, nonlinear optical materials, radiopharmaceuticals, reagents for CO-reduction chemistry and photopolymerization (Kumar *et al.*, 2010 ▸). As a consequence, among organometallic complexes, tri­carbonyl­rhenium(I) compounds have received considerable attention. Facile synthesis and previously available knowledge of their photophysics (Stufkens & Vlcek, 1998 ▸) encouraged us to design new photo-active carbonyl­rhenium complexes as CO-donating mol­ecules. Photo-active metal–carbonyl complexes (photoCORMs) have been utilized as more controllable CO donors to exploit various salutary effects in mammalian pathophysiology when administered in moderate concentrations (Gonzalez & Mascharak, 2014 ▸; Romao *et al.*, 2012 ▸; Schatzschneider, 2015 ▸). We (Carrington *et al.*, 2016 ▸) and others (Zobi *et al.*, 2012 ▸) have shown applications of rhenium carbonyl-based photoCORMs towards the eradication of aggressive malignant cells, as well as oxidatively damaged cell restoration through light-induced CO delivery. Along the line of developing metal–carbonyl complex-based photoCORMs (Chakraborty *et al.*, 2014 ▸), we report herein the synthesis and structural characterization of a carbonyl­rhenium complex, [ReCl(pyAm)(CO)_3_], where pyAm is 1-[(pyridin-2-yl­methyl­idene)amino]­adamantane. In this design of pyAm ligand, the adamantyl moiety has been included beacuse of its well-known pharmacokinetic properties (Wanka *et al.*, 2013 ▸).

## Structural commentary   

**Figure 1:**
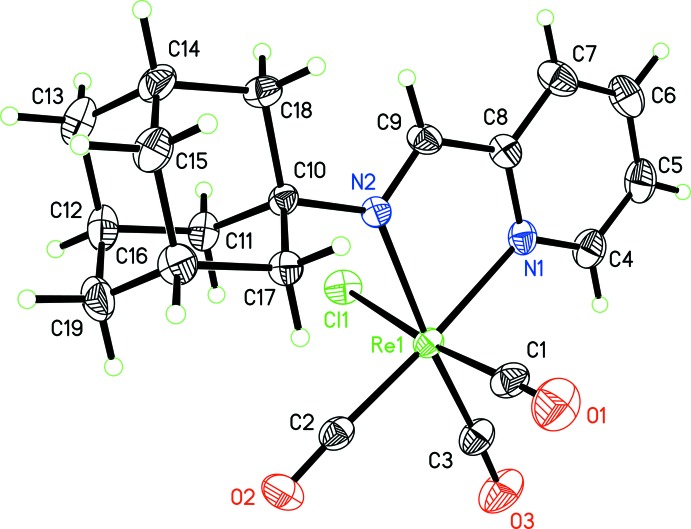
The mol­ecular structure of the title complex. Displacement ellipsoids correspond to 50% probability levels.

The mol­ecular structure of the title complex is shown in Fig. 1 ▸. The coordination geometry of Re^I^ in the complex is distorted octa­hedral (Table 1 ▸). The pyAm ligand binds the metal in a bidenate fashion, while the three CO ligands reside in a *facial* disposition. The distortion from ideal values is reflected by the N1—Re1—N2 bite angle of 75.41 (9)°. The sixth site is occupied by a chloride ligand. The equatorial plane composed of atoms N1, N2, C2 and C3 is satisfactorily planar, with a mean deviation of 0.034 Å. In this complex, the chelate ring composed of atoms Re1, N1, C8, C9 and N2 is almost planar, with a mean deviation of 0.007 Å. The Re—Cl bond is considerably longer [1.963 (4) Å] compared to the other two Re—C bonds [1.918 (4) and 1.920 (3) Å], which can be attributed to the *trans*-labilizing effect arising from the chloride ligand across this bond.
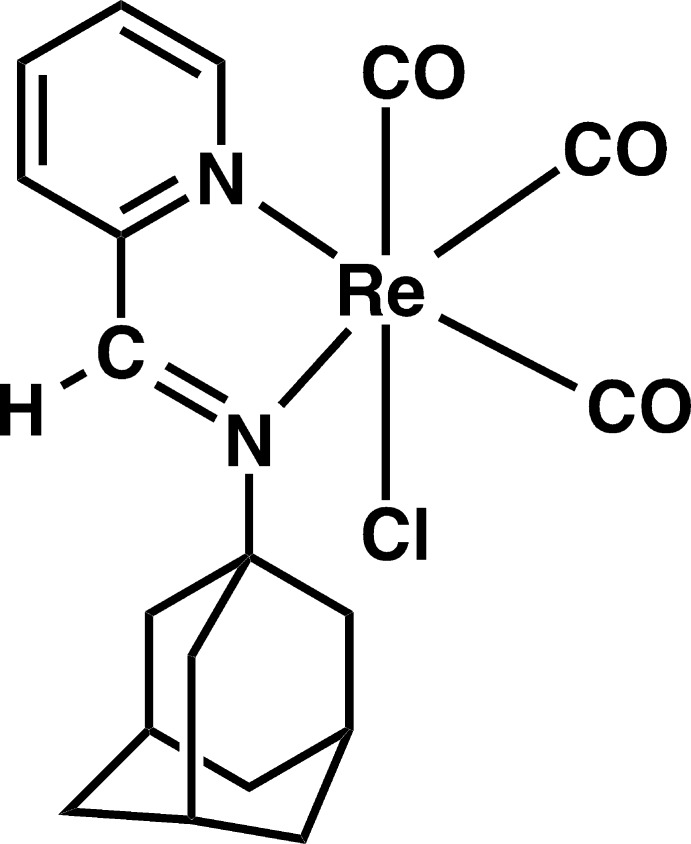


## Supra­molecular features   

**Figure 2:**
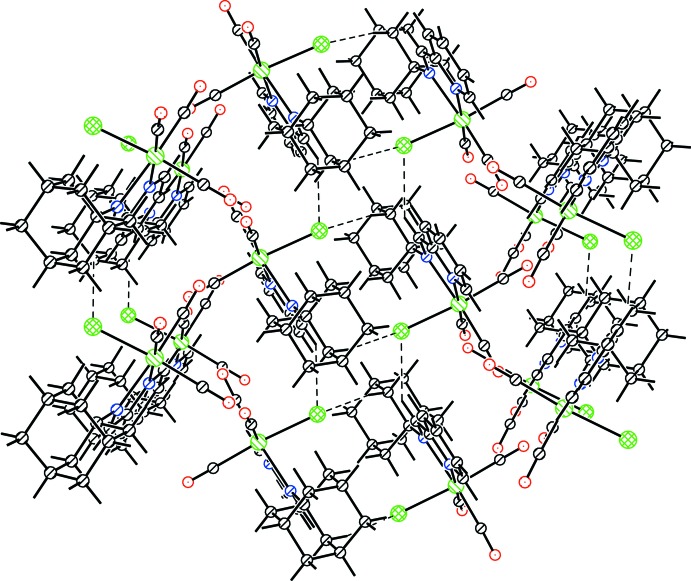
Packing pattern of the title complex, showing the C—H⋯Cl inter­actions.

**Figure 3:**
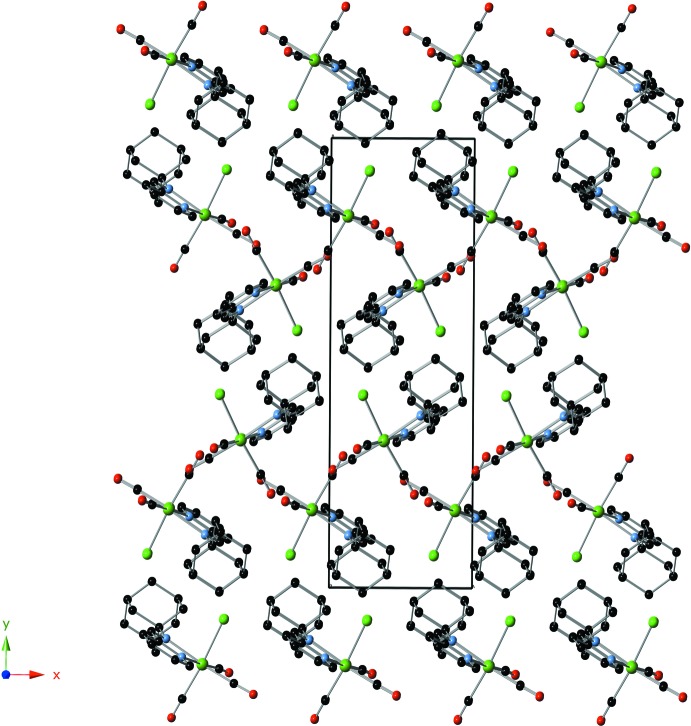
Packing diagram of the title complex along the *c* axis.

The crystal packing of the title complex reveals few nonclassical hydrogen-bonding inter­actions of the C—H⋯Cl type (Table 2 ▸ and Fig. 2 ▸), leading to a three-dimensional network structure. The arrangement of mol­ecules along the *c* axis is shown in Fig. 3 ▸.

## Database survey   

A search of the Cambridge Structural Database (Groom *et al.*, 2016 ▸) revealed only a few structurally similar complexes, with a general formula of [ReCl(py*R*)(CO)_3_], where *R* represents substituted or unsubstituted aromatic amines. The complex [ReCl(2-PP)(CO)_3_] [where 2-PP = *N*-(pyridin-2-yl­methyl­idene)aniline] has space-group symmetry *P*2_1_/*n* (Dominey *et al.*, 1991 ▸) and exhibits comparable metric parameters as the title complex. However, careful scrunity reveals that in this case the *trans*-influence of the chloride ligand is not reflected as in the title complex. Later, the same complex was found to adopt also triclinic symmetry in the *P* 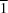 space group (Hasheminasab *et al.*, 2014 ▸). Another complex, [ReCl(*L* ^1^)(CO)_3_] {where *L* ^1^ = 4-[(pyridin-2-yl­methyl­idene)amino]­phenol} has *P*2_1_/*n* space-group symmetry, with unit-cell dimensions close to those of [ReCl(2-PP)(CO)_3_] (Liu & Heinze, 2010 ▸). In another report, two rhenium complexes of the general formula [ReCl(pyca-C_6_H_4_OH)(CO)_3_] (where pyca = pyridine-2-carbaldehyde­imine) were structurally characterized (Chanawanno *et al.*, 2013 ▸). In this case, the two complexes can be differentiated on the basis of the position of the –OH group on the arene ring. The complex with the –OH group at the *meta* position was described in the *P*2_1_/*c* space group, while that with the –OH group in the *ortho* position of the arene ring was described in the setting *P*2_1_/*n*. In a relatively recent report, another rhenium complex, namely [ReCl(pyca-2,6-iPr_2_C_6_H_3_)(CO)_3_], was synthesized (*C*2/*c*; Kianfar *et al.*, 2015 ▸). However, no such rhenium complex incorporating an aliphatic amine in the Schiff base ligand has been structurally characterized so far.

## Synthesis and crystallization   

A slurry of 50 mg (0.138 mmol) of [ReCl(CO)_5_] and 33 mg of pyAm (0.138 mmol) were added in a mixture of 15 ml of methanol and 5 ml of chloro­form and allowed to reflux for 24 h. After this time, the reaction mixture was allowed to cool to room temperature, whereupon an orange precipitate was observed. The orange solid was collected by filtration and dried under vacuum to obtain 44.2 mg (55%) of the title complex. Single crystals were obtained by layering hexa­nes over a di­chloro­methane solution.

## Refinement   

Crystal data, data collection and structure refinement details are summarized in Table 3 ▸. H atoms were included in calculated positions on the C atoms to which they are bonded, with C—H = 0.93 Å and *U*_iso_(H) = 1.2*U*_eq_(C). One reflection (*i.e.* 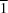
01) was removed from the refinement because it was partly obscured by the beam stop.

## Supplementary Material

Crystal structure: contains datablock(s) I. DOI: 10.1107/S205698901601255X/wm5310sup1.cif

Structure factors: contains datablock(s) I. DOI: 10.1107/S205698901601255X/wm5310Isup2.hkl

CCDC reference: 1497515

Additional supporting information: crystallographic information; 3D view; checkCIF report

## Figures and Tables

**Table 1:** Selected geometric parameters (Å, °)

Re1—C2	1.918 (4)	Re1—N1	2.175 (3)
Re1—C3	1.920 (3)	Re1—N2	2.213 (2)
Re1—C1	1.963 (4)	Re1—Cl1	2.4700 (8)
			
C2—Re1—C3	86.46 (14)	C1—Re1—N2	92.65 (11)
C2—Re1—C1	88.36 (14)	N1—Re1—N2	75.41 (9)
C3—Re1—C1	92.35 (14)	C2—Re1—Cl1	96.08 (9)
C2—Re1—N1	176.25 (11)	C3—Re1—Cl1	90.98 (11)
C3—Re1—N1	97.12 (13)	C1—Re1—Cl1	174.61 (10)
C1—Re1—N1	92.59 (12)	N1—Re1—Cl1	82.78 (7)
C2—Re1—N2	100.93 (11)	N2—Re1—Cl1	83.53 (6)
C3—Re1—N2	171.19 (12)		

**Table 2:** Hydrogen-bond geometry (Å, °)

*D*—H⋯*A*	*D*—H	H⋯*A*	*D*⋯*A*	*D*—H⋯*A*
C9—H9⋯Cl1^i^	0.93	2.76	3.523 (3)	140
C7—H7⋯Cl1^i^	0.93	2.92	3.662 (4)	137
C18—H18*A*⋯Cl1^ii^	0.97	2.74	3.701 (3)	170

Symmetry codes: (i) 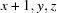; (ii) 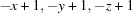.

**Table 3:** Experimental details

Crystal data	
Chemical formula	[ReCl(C_16_H_20_N_2_)(CO)_3_]
*M*_r_	546.02
Crystal system, space group	Monoclinic, *P*2_1_/*n*
Temperature (K)	273
*a*, *b*, *c* (Å)	6.9550 (6), 21.7483 (19), 12.4482 (11)
β (°)	94.509 (1)
*V* (Å^3^)	1877.1 (3)
*Z*	4
Radiation type	Mo *K*α
μ (mm^−1^)	6.64
Crystal size (mm)	0.15 × 0.10 × 0.04

Data collection	
Diffractometer	Bruker APEXII CCD
Absorption correction	Multi-scan (*SADABS*; Bruker, 2012 ▸)
*T*_min_, *T*_max_	0.496, 0.745
No. of measured, independent and observed [*I* > 2σ(*I*)] reflections	20008, 4728, 4045
*R*_int_	0.027
(sin θ/λ)_max_ (Å^−1^)	0.683

Refinement	
*R*[*F* ^2^ > 2σ(*F* ^2^)], *wR*(*F* ^2^), *S*	0.023, 0.050, 1.05
No. of reflections	4728
No. of parameters	235
H-atom treatment	H-atom parameters constrained
Δρ_max_, Δρ_min_ (e Å^−3^)	0.79, −0.55

Computer programs: *APEX2* and *SAINT* (Bruker, 2012 ▸), *SHELXT* (Sheldrick, 2015*a* ▸), *SHELXL2014* (Sheldrick, 2015*b* ▸), *XP* in *SHELXTL* (Sheldrick, 2008 ▸), *CrystalMaker* (Palmer, 2014 ▸) and *publCIF* (Westrip, 2010 ▸).

## supplementary crystallographic information

### Crystal data

**Table:**

[ReCl(C_16_H_20_N_2_)(CO)_3_]	*F*(000) = 1056
*M**_r_* = 546.02	*D*_x_ = 1.932 Mg m^−^^3^
Monoclinic, *P*2_1_/*n*	Mo *K*α radiation, λ = 0.71073 Å
*a* = 6.9550 (6) Å	Cell parameters from 8126 reflections
*b* = 21.7483 (19) Å	θ = 2.5–24.1°
*c* = 12.4482 (11) Å	µ = 6.64 mm^−^^1^
β = 94.509 (1)°	*T* = 273 K
*V* = 1877.1 (3) Å^3^	Plate, yellow
*Z* = 4	0.15 × 0.10 × 0.04 mm

### Data collection

**Table:**

Bruker APEXII CCD diffractometer	4045 reflections with *I* > 2σ(*I*)
ω scans	*R*_int_ = 0.027
Absorption correction: multi-scan (*SADABS*; Bruker, 2012)	θ_max_ = 29.1°, θ_min_ = 2.5°
*T*_min_ = 0.496, *T*_max_ = 0.745	*h* = −9→9
20008 measured reflections	*k* = −29→29
4728 independent reflections	*l* = −16→16

### Refinement

**Table:**

Refinement on *F*^2^	0 restraints
Least-squares matrix: full	Hydrogen site location: inferred from neighbouring sites
*R*[*F*^2^ > 2σ(*F*^2^)] = 0.023	H-atom parameters constrained
*wR*(*F*^2^) = 0.050	*w* = 1/[σ^2^(*F*_o_^2^) + (0.0213*P*)^2^ + 0.918*P*] where *P* = (*F*_o_^2^ + 2*F*_c_^2^)/3
*S* = 1.05	(Δ/σ)_max_ = 0.002
4728 reflections	Δρ_max_ = 0.79 e Å^−^^3^
235 parameters	Δρ_min_ = −0.55 e Å^−^^3^

### Special details

**Table:**

Geometry. All esds (except the esd in the dihedral angle between two l.s. planes) are estimated using the full covariance matrix. The cell esds are taken into account individually in the estimation of esds in distances, angles and torsion angles; correlations between esds in cell parameters are only used when they are defined by crystal symmetry. An approximate (isotropic) treatment of cell esds is used for estimating esds involving l.s. planes.

### Fractional atomic coordinates and isotropic or equivalent isotropic displacement parameters (Å^2^)

**Table:**

	*x*	*y*	*z*	*U*_iso_*/*U*_eq_	
Re1	0.37969 (2)	0.32771 (2)	0.53456 (2)	0.03239 (5)	
Cl1	0.23110 (12)	0.42838 (4)	0.48815 (7)	0.0476 (2)	
N2	0.6343 (3)	0.38424 (11)	0.58745 (18)	0.0298 (5)	
O2	0.1930 (4)	0.30895 (12)	0.7472 (2)	0.0568 (7)	
N1	0.5242 (4)	0.34377 (12)	0.3884 (2)	0.0371 (6)	
O3	0.0223 (4)	0.26163 (13)	0.4307 (3)	0.0742 (9)	
O1	0.5930 (4)	0.20826 (14)	0.5848 (2)	0.0729 (8)	
C17	0.7065 (4)	0.35397 (14)	0.7773 (2)	0.0335 (6)	
H17A	0.5911	0.3288	0.7708	0.040*	
H17B	0.8147	0.3288	0.7598	0.040*	
C2	0.2629 (5)	0.31762 (14)	0.6679 (3)	0.0399 (7)	
C8	0.6851 (4)	0.37871 (15)	0.4017 (2)	0.0374 (7)	
C10	0.6832 (4)	0.40873 (13)	0.6989 (2)	0.0296 (6)	
C1	0.5193 (5)	0.25045 (17)	0.5650 (3)	0.0444 (8)	
C9	0.7374 (4)	0.39986 (15)	0.5113 (2)	0.0374 (7)	
H9	0.8454	0.4246	0.5257	0.045*	
C7	0.7933 (5)	0.39368 (18)	0.3160 (3)	0.0509 (9)	
H7	0.9039	0.4176	0.3269	0.061*	
C4	0.4683 (6)	0.32441 (16)	0.2888 (3)	0.0501 (9)	
H4	0.3568	0.3008	0.2787	0.060*	
C3	0.1587 (5)	0.28552 (16)	0.4682 (3)	0.0472 (8)	
C18	0.8699 (4)	0.44667 (15)	0.7101 (2)	0.0384 (7)	
H18A	0.8594	0.4813	0.6609	0.046*	
H18B	0.9777	0.4215	0.6919	0.046*	
C14	0.9052 (5)	0.47005 (15)	0.8271 (3)	0.0444 (8)	
H14	1.0239	0.4945	0.8337	0.053*	
C15	0.9266 (5)	0.41588 (16)	0.9048 (3)	0.0460 (8)	
H15A	0.9496	0.4307	0.9782	0.055*	
H15B	1.0352	0.3906	0.8881	0.055*	
C5	0.5687 (6)	0.33785 (17)	0.2005 (3)	0.0559 (10)	
H5	0.5250	0.3237	0.1325	0.067*	
C19	0.5695 (5)	0.41766 (18)	0.9200 (3)	0.0511 (9)	
H19A	0.4522	0.3934	0.9129	0.061*	
H19B	0.5884	0.4321	0.9939	0.061*	
C16	0.7408 (5)	0.37820 (15)	0.8932 (2)	0.0423 (7)	
H16	0.7528	0.3433	0.9430	0.051*	
C11	0.5154 (5)	0.44984 (15)	0.7271 (2)	0.0392 (7)	
H11A	0.5044	0.4848	0.6785	0.047*	
H11B	0.3957	0.4268	0.7188	0.047*	
C6	0.7330 (6)	0.37222 (18)	0.2143 (3)	0.0564 (10)	
H6	0.8038	0.3811	0.1558	0.068*	
C12	0.5508 (5)	0.47239 (17)	0.8435 (3)	0.0505 (9)	
H12	0.4420	0.4980	0.8616	0.061*	
C13	0.7357 (6)	0.51060 (17)	0.8544 (3)	0.0563 (10)	
H13A	0.7576	0.5261	0.9274	0.068*	
H13B	0.7239	0.5455	0.8057	0.068*	

### Atomic displacement parameters (Å^2^)

**Table:**

	*U*^11^	*U*^22^	*U*^33^	*U*^12^	*U*^13^	*U*^23^
Re1	0.03066 (7)	0.03249 (7)	0.03334 (7)	−0.00218 (5)	−0.00171 (5)	−0.00180 (5)
Cl1	0.0399 (5)	0.0493 (5)	0.0525 (5)	0.0033 (4)	−0.0035 (4)	0.0066 (4)
N2	0.0258 (12)	0.0349 (13)	0.0281 (12)	0.0010 (10)	−0.0019 (9)	−0.0014 (10)
O2	0.0540 (16)	0.0645 (16)	0.0542 (16)	−0.0045 (13)	0.0188 (13)	0.0064 (13)
N1	0.0388 (15)	0.0427 (15)	0.0293 (13)	0.0026 (11)	−0.0009 (11)	−0.0061 (11)
O3	0.0520 (17)	0.0655 (18)	0.100 (2)	−0.0128 (14)	−0.0233 (16)	−0.0201 (16)
O1	0.067 (2)	0.065 (2)	0.085 (2)	0.0119 (16)	−0.0044 (16)	−0.0020 (16)
C17	0.0331 (16)	0.0357 (15)	0.0311 (15)	0.0000 (13)	−0.0009 (12)	0.0012 (12)
C2	0.0325 (17)	0.0354 (17)	0.051 (2)	−0.0013 (13)	−0.0018 (15)	−0.0034 (14)
C8	0.0340 (17)	0.0462 (18)	0.0316 (15)	0.0040 (13)	−0.0001 (12)	0.0014 (13)
C10	0.0320 (15)	0.0315 (15)	0.0245 (14)	−0.0029 (12)	−0.0028 (11)	−0.0018 (11)
C1	0.048 (2)	0.0429 (19)	0.0407 (18)	−0.0109 (16)	−0.0049 (15)	−0.0019 (15)
C9	0.0267 (16)	0.0519 (19)	0.0333 (16)	−0.0056 (13)	0.0000 (12)	0.0000 (13)
C7	0.043 (2)	0.074 (3)	0.0352 (18)	−0.0016 (18)	0.0046 (15)	0.0082 (17)
C4	0.059 (2)	0.053 (2)	0.0364 (18)	−0.0015 (17)	−0.0052 (16)	−0.0083 (15)
C3	0.043 (2)	0.0428 (19)	0.054 (2)	−0.0011 (15)	−0.0085 (16)	−0.0058 (15)
C18	0.0365 (17)	0.0397 (17)	0.0381 (17)	−0.0081 (13)	−0.0027 (13)	0.0039 (13)
C14	0.046 (2)	0.0445 (19)	0.0405 (18)	−0.0113 (15)	−0.0074 (15)	−0.0069 (14)
C15	0.047 (2)	0.054 (2)	0.0351 (17)	−0.0024 (16)	−0.0121 (15)	−0.0053 (15)
C5	0.076 (3)	0.059 (2)	0.0315 (18)	0.008 (2)	−0.0020 (17)	−0.0086 (16)
C19	0.052 (2)	0.073 (3)	0.0290 (16)	−0.0026 (18)	0.0039 (15)	−0.0115 (16)
C16	0.049 (2)	0.0476 (19)	0.0294 (16)	−0.0015 (15)	−0.0048 (14)	0.0050 (13)
C11	0.0377 (18)	0.0419 (18)	0.0370 (17)	0.0085 (14)	−0.0038 (13)	−0.0068 (13)
C6	0.064 (3)	0.074 (3)	0.0324 (18)	0.010 (2)	0.0105 (17)	0.0049 (17)
C12	0.049 (2)	0.057 (2)	0.0452 (19)	0.0102 (17)	−0.0002 (16)	−0.0187 (17)
C13	0.069 (3)	0.047 (2)	0.050 (2)	0.0017 (18)	−0.0100 (18)	−0.0169 (17)

### Geometric parameters (Å, º)

**Table:**

Re1—C2	1.918 (4)	C4—H4	0.9300
Re1—C3	1.920 (3)	C18—C14	1.545 (4)
Re1—C1	1.963 (4)	C18—H18A	0.9700
Re1—N1	2.175 (3)	C18—H18B	0.9700
Re1—N2	2.213 (2)	C14—C15	1.524 (5)
Re1—Cl1	2.4700 (8)	C14—C13	1.532 (5)
N2—C9	1.279 (4)	C14—H14	0.9800
N2—C10	1.500 (3)	C15—C16	1.527 (4)
O2—C2	1.149 (4)	C15—H15A	0.9700
N1—C4	1.338 (4)	C15—H15B	0.9700
N1—C8	1.352 (4)	C5—C6	1.365 (5)
O3—C3	1.149 (4)	C5—H5	0.9300
O1—C1	1.070 (4)	C19—C12	1.524 (5)
C17—C16	1.537 (4)	C19—C16	1.526 (5)
C17—C10	1.541 (4)	C19—H19A	0.9700
C17—H17A	0.9700	C19—H19B	0.9700
C17—H17B	0.9700	C16—H16	0.9800
C8—C7	1.392 (4)	C11—C12	1.532 (4)
C8—C9	1.458 (4)	C11—H11A	0.9700
C10—C11	1.533 (4)	C11—H11B	0.9700
C10—C18	1.535 (4)	C6—H6	0.9300
C9—H9	0.9300	C12—C13	1.528 (5)
C7—C6	1.383 (5)	C12—H12	0.9800
C7—H7	0.9300	C13—H13A	0.9700
C4—C5	1.379 (5)	C13—H13B	0.9700
			
C2—Re1—C3	86.46 (14)	C10—C18—H18B	109.8
C2—Re1—C1	88.36 (14)	C14—C18—H18B	109.8
C3—Re1—C1	92.35 (14)	H18A—C18—H18B	108.2
C2—Re1—N1	176.25 (11)	C15—C14—C13	110.1 (3)
C3—Re1—N1	97.12 (13)	C15—C14—C18	110.1 (3)
C1—Re1—N1	92.59 (12)	C13—C14—C18	109.3 (3)
C2—Re1—N2	100.93 (11)	C15—C14—H14	109.1
C3—Re1—N2	171.19 (12)	C13—C14—H14	109.1
C1—Re1—N2	92.65 (11)	C18—C14—H14	109.1
N1—Re1—N2	75.41 (9)	C14—C15—C16	108.4 (3)
C2—Re1—Cl1	96.08 (9)	C14—C15—H15A	110.0
C3—Re1—Cl1	90.98 (11)	C16—C15—H15A	110.0
C1—Re1—Cl1	174.61 (10)	C14—C15—H15B	110.0
N1—Re1—Cl1	82.78 (7)	C16—C15—H15B	110.0
N2—Re1—Cl1	83.53 (6)	H15A—C15—H15B	108.4
C9—N2—C10	119.4 (2)	C6—C5—C4	119.2 (3)
C9—N2—Re1	114.15 (19)	C6—C5—H5	120.4
C10—N2—Re1	126.21 (17)	C4—C5—H5	120.4
C4—N1—C8	117.9 (3)	C12—C19—C16	109.4 (3)
C4—N1—Re1	127.2 (2)	C12—C19—H19A	109.8
C8—N1—Re1	114.88 (19)	C16—C19—H19A	109.8
C16—C17—C10	109.3 (2)	C12—C19—H19B	109.8
C16—C17—H17A	109.8	C16—C19—H19B	109.8
C10—C17—H17A	109.8	H19A—C19—H19B	108.2
C16—C17—H17B	109.8	C19—C16—C15	110.3 (3)
C10—C17—H17B	109.8	C19—C16—C17	109.4 (3)
H17A—C17—H17B	108.3	C15—C16—C17	109.9 (3)
O2—C2—Re1	177.1 (3)	C19—C16—H16	109.1
N1—C8—C7	122.0 (3)	C15—C16—H16	109.1
N1—C8—C9	115.8 (3)	C17—C16—H16	109.1
C7—C8—C9	122.2 (3)	C12—C11—C10	109.6 (2)
N2—C10—C11	107.3 (2)	C12—C11—H11A	109.8
N2—C10—C18	113.8 (2)	C10—C11—H11A	109.8
C11—C10—C18	108.6 (2)	C12—C11—H11B	109.8
N2—C10—C17	108.5 (2)	C10—C11—H11B	109.8
C11—C10—C17	110.5 (2)	H11A—C11—H11B	108.2
C18—C10—C17	108.2 (2)	C5—C6—C7	119.4 (3)
O1—C1—Re1	177.7 (4)	C5—C6—H6	120.3
N2—C9—C8	119.8 (3)	C7—C6—H6	120.3
N2—C9—H9	120.1	C19—C12—C13	109.8 (3)
C8—C9—H9	120.1	C19—C12—C11	109.9 (3)
C6—C7—C8	118.7 (3)	C13—C12—C11	109.2 (3)
C6—C7—H7	120.7	C19—C12—H12	109.3
C8—C7—H7	120.7	C13—C12—H12	109.3
N1—C4—C5	122.9 (4)	C11—C12—H12	109.3
N1—C4—H4	118.5	C12—C13—C14	108.9 (3)
C5—C4—H4	118.5	C12—C13—H13A	109.9
O3—C3—Re1	177.5 (3)	C14—C13—H13A	109.9
C10—C18—C14	109.5 (2)	C12—C13—H13B	109.9
C10—C18—H18A	109.8	C14—C13—H13B	109.9
C14—C18—H18A	109.8	H13A—C13—H13B	108.3
			
C4—N1—C8—C7	−1.2 (5)	C10—C18—C14—C15	60.7 (3)
Re1—N1—C8—C7	−179.4 (3)	C10—C18—C14—C13	−60.3 (3)
C4—N1—C8—C9	178.6 (3)	C13—C14—C15—C16	60.5 (3)
Re1—N1—C8—C9	0.3 (3)	C18—C14—C15—C16	−60.1 (4)
C9—N2—C10—C11	114.9 (3)	N1—C4—C5—C6	0.2 (6)
Re1—N2—C10—C11	−59.7 (3)	C12—C19—C16—C15	59.8 (3)
C9—N2—C10—C18	−5.3 (4)	C12—C19—C16—C17	−61.2 (4)
Re1—N2—C10—C18	−179.86 (19)	C14—C15—C16—C19	−60.1 (3)
C9—N2—C10—C17	−125.8 (3)	C14—C15—C16—C17	60.6 (3)
Re1—N2—C10—C17	59.7 (3)	C10—C17—C16—C19	59.6 (3)
C16—C17—C10—N2	−175.5 (2)	C10—C17—C16—C15	−61.6 (3)
C16—C17—C10—C11	−58.2 (3)	N2—C10—C11—C12	175.8 (3)
C16—C17—C10—C18	60.6 (3)	C18—C10—C11—C12	−60.7 (3)
C10—N2—C9—C8	−177.0 (3)	C17—C10—C11—C12	57.8 (3)
Re1—N2—C9—C8	−1.8 (4)	C4—C5—C6—C7	−1.1 (6)
N1—C8—C9—N2	1.0 (4)	C8—C7—C6—C5	0.9 (5)
C7—C8—C9—N2	−179.2 (3)	C16—C19—C12—C13	−59.3 (4)
N1—C8—C7—C6	0.3 (5)	C16—C19—C12—C11	61.0 (4)
C9—C8—C7—C6	−179.5 (3)	C10—C11—C12—C19	−59.0 (4)
C8—N1—C4—C5	0.9 (5)	C10—C11—C12—C13	61.6 (4)
Re1—N1—C4—C5	178.9 (3)	C19—C12—C13—C14	59.6 (4)
N2—C10—C18—C14	179.4 (2)	C11—C12—C13—C14	−61.1 (4)
C11—C10—C18—C14	60.0 (3)	C15—C14—C13—C12	−60.6 (3)
C17—C10—C18—C14	−60.0 (3)	C18—C14—C13—C12	60.5 (4)

### Hydrogen-bond geometry (Å, º)

**Table:**

*D*—H···*A*	*D*—H	H···*A*	*D*···*A*	*D*—H···*A*
C9—H9···Cl1^i^	0.93	2.76	3.523 (3)	140
C7—H7···Cl1^i^	0.93	2.92	3.662 (4)	137
C18—H18*A*···Cl1^ii^	0.97	2.74	3.701 (3)	170

Symmetry codes: (i) *x*+1, *y*, *z*; (ii) −*x*+1, −*y*+1, −*z*+1.
